# Impact of Engineered Particle Size Distribution of Ordinary Portland Cement on Slag Supplemented Cement: A Comparative Performance Analysis With Commercial Portland Slag Cement

**DOI:** 10.1155/tswj/5442750

**Published:** 2025-04-25

**Authors:** Rajan Suresh Kamble, Krishnasetty Govindaraja Guptha, Ashish Kumar Nayak, Jagadish Vengala

**Affiliations:** ^1^Department of Civil Engineering, Goa College of Engineering (Affiliated to Goa University), Ponda, Goa, India; ^2^Department of Civil Engineering, Prasad V. Potluri Siddhartha Institute of Technology, Vijayawada, Andhra Pradesh, India

## Abstract

This study explores the critical interplay between particle size distribution (PSD) and Bogue's compounds, highlighting their essential roles in enhancing the early strength of cement while ensuring sustainability. The research explores experimental procedures by subjecting commercial ordinary Portland cement (C-OPC) to a grinding process, resulting in a specific fineness of 5390 cm^2^/g, referred to as the “stimulator (S)”. Blending this stimulator with standard C-OPC at a precisely calibrated 45% weight ratio (referred to as ‘S45') demonstrates a refined approach to optimizing the PSD of the mixture. The results confirm the superior properties of the resulting slag cement blend, with engineered PSD serving as the central driver of these improvements. To broaden the study's scope, ground granulated blast furnace slag (GGBFS) is introduced as a supplementary cementitious material (SCM) and strategically combined with S45 in varying proportions. This systematic approach shows that the optimized blend of S45 and GGBFS outperforms commercial Portland slag cement (Com-PSC) and ushers in a new paradigm in cement formulation using SCMs. The findings underscore the significance of PSD in enhancing both the initial and long-term compressive strength of cement, with performance improvements evaluated over a period of up to 365 days. Importantly, the optimized approach enables the production of more sustainable cement without increasing production costs. This not only reduces the carbon footprint but also promotes a safer and more environmentally friendly industry. The research highlights a practical pathway for cement manufacturers to enhance the performance of slag cement, though the quality of slag remains dependent on its source. Future research aimed at developing comprehensive guidelines will provide valuable insights to further advance sustainable cement production.

## 1. Introduction

Supplementary cementitious material (SCM) blended cements have demonstrated superior performance in terms of both durability [1–3] and strength gain over time [4–6] when compared to ordinary Portland cement (OPC). Furthermore, a notable reduction in the exothermic heat released during hydration [7] is also a well-established fact observed in SCM-blended cement. Also, a considerable reduction in carbon dioxide emission [8] associated with the production and application of SCM-blended cement, aligning with environmental sustainability goals, is achieved by utilizing industrial wastes [9]. However, significant drawbacks in substituting OPC with SCM have been reported, such as low initial strength values [10] with retarded gain [11], particularly in the case of slag-blended cements currently available in the market. High water requirements [12] for the hydration process, along with delayed setting times [13], are also noticed in such cements. This has resulted in limiting SCM substitution levels in OPC [14]. The existed literature (up to 2022) gap in this regard is addressed by this work.

While the literature has extensively explored the impact of maximizing SCM content in OPC blends, there is a noticeable gap in comprehensive studies that incorporate particle size distribution (PSD) of OPC as a key factor influencing the overall performance of SCM-blended cement, especially following the seminal work of Chakurkar and Guptha [15]. Their groundbreaking research reported a significant enhancement in early strength, with lower consistency values not previously documented. The pronounced influence on both hydrated and unhydrated cement's low heat of hydration, coupled with improved workability of cement and concrete, further underscores the transformative potential of blended cement. Notably, there is a scarcity of research addressing the engineered distribution of particle sizes in blends of the stimulator and slag, a unique aspect not yet explored by any researcher. The present study, building upon the pioneering work of Chakurkar and Guptha [15], thus fills this crucial research gap by optimizing blend proportions in accordance with engineered PSD, following codal provisions.

This study also contributes to reducing the environmental footprint of the construction industry, particularly in terms of resource efficiency and waste reduction. Measures were taken to ensure the long-term durability and resilience of cement blends incorporating ground granulated blast furnace slag (GGBFS) as an SCM. The results of this research align with or challenge existing standards and regulations regarding cement production and use, especially in relation to environmental protection and sustainable development goals. Additionally, the study provides insights into the economic viability of incorporating engineered PSD and supplementary materials like GGBFS into cement formulations, considering potential cost implications for the construction industry. The study also identifies the optimized combination of S45 and GGBFS to address specific environmental challenges associated with traditional cement formulations, such as carbon emissions and resource depletion. The findings of this study, in collaboration with a cement-producing industry, are expected to influence future research directions and industry practices in the field of environmental engineering, particularly in the context of sustainable construction materials and practices.

The proposed methodology does not require any additional facilities or machinery in the production line from an industry perspective. The product has been tested and complies with all relevant codal provisions.

## 2. Experimental Studies

### 2.1. Materials

In devising slag-blended combinations, commercial OPC (C-OPC) and GGBFS adhering to BIS 269 [16] and BIS 455 [17] serve as essential constituents, respectively. This study primarily involves introducing a component of cement termed “stimulator (S)” (Chakurkar and Guptha [15]), characterized by an engineered PSD of C-OPC. Their work involved the optimized micronization of C-OPC by breaking it down into finer particles and amalgamating stimulator (S) with C-OPC to produce engineered cement. They demonstrated the significantly enhanced physio-mechanical performance of the resultant cement, attributing it to the redistribution of particle sizes in the derived cement matrix. They concluded that incorporating 40%–50% of the Stimulator in C-OPC was the optimized proportion that resulted in a balanced macro–microparticle distribution in the modified cement, leading to early strength gain, improved consistency, and enhanced durability against permeability and acid attack.

The essential properties of C-OPC, S, and GGBFS are presented in Table 1 for comprehensive reference.

#### 2.1.1. Stimulator

Micronization of the C-OPC was carried out using jet-milling to derive the stimulator, following the procedure established by Chakurkar and Guptha [15]. The properties are detailed in Table 1.

#### 2.1.2. Slag Combinations

This study primarily aims to evaluate the impact of PSD-engineered C-OPC, as articulated by Chakurkar and Guptha [15], on slag-supplemented blended cement, examining its physio-mechanical properties and comparing the results with commercial Portland slag cement (Com-PSC). Chakurkar and Guptha [15] optimized the proportion of C-OPC to stimulator at 55% and 45%, respectively, by weight. This blend is designated as ‘S45.' Slag combinations consisting of S45 and GGBFS were meticulously formulated in accordance with the stipulations outlined in BIS 12089 [18]. BIS 455-2015 (reaffirmed in 2020) [17] specifies slag content ranging from 25% to 70%. Accordingly, the specifications for the combinations were followed and addressed in the analysis.  Table 2 provides the composition of the blends selected for this study. The specific impacts of these combinations on performance and sustainability are discussed. Notably, these investigations were conducted while maintaining the chemical composition with marginal variations, accounting for randomization of particles to ensure a focused examination of the influence of PSD on the identified parameters.

### 2.2. Tests Performed on Slag Combinations

Quality assessment of the combinations proposed in Table 2 was accomplished through the following tests.

#### 2.2.1. X-Ray Fluorescence

X-ray fluorescence spectrometric analyses of slag combinations and Com-PSC were conducted using equipment (ARL 9900, Thermo Fisher Scientific) in accordance with BIS 12803 [19]. The results are presented in Table 3.

#### 2.2.2. PSD by Laser Diffraction

**Figure 1:**
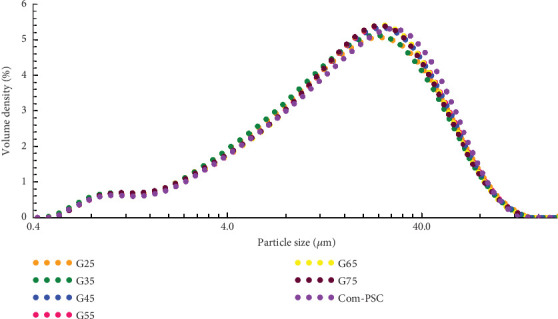
Particle size distribution (PSD) of slag combinations and commercial PSC.

The PSD of slag combinations and Com-PSC was determined using the laser diffraction method with equipment called the Mastersizer 3000 (Malvern Panalytical), following ISO 13320 [20] and ASTM E-3340 [21] specifications. Measurements of angular variation under the intensity of scattered light were recorded and are presented in Table 4.  Figure 1 illustrates the PSD of the slag combinations and Com-PSC, providing a clear representation of the varying particle sizes within the combinations.

#### 2.2.3. Standard Consistency (SC)

The SC test was conducted for all slag combinations and Com-PSC in accordance with IS 4031-4 [22]. Potable water complying with IS 456:2000 [23] was used throughout the experimental work. The test results are presented in Table 5.

#### 2.2.4. Initial and Final Setting Times

The initial and final setting times for all slag combinations and Com-PSC were meticulously determined in accordance with the specifications outlined in BIS 4031-5 [24]. This assessment involved measuring the time intervals from the addition of water to the powder until specific setting criteria were achieved. The values obtained for the setting times are presented in Table 5.

#### 2.2.5. Compressive Strength

Following the guidelines specified in BIS 4031-6 [25], mortar cubes were cast for various slag combinations using standard sand conforming to BIS 650 (2018) [26] and with mechanical compaction in accordance with BIS 10080 (2018) [27]. These cubes were tested using an automatic compression testing machine (model no. AIM 314E-MU-1) manufactured by Aimil Ltd. at curing ages of 3, 7, and 28 days to assess their compressive strength. The results are presented in Table 5 for comparison with Com-PSC.

#### 2.2.6. Durability Tests

After evaluating the tests results of all slag combinations listed in Sections 2.2.1–2.2.5, the optimized blend of S45 and GGBFS, designated as G45, was further subjected to durability tests on concrete cubes. These tests included (a) rapid chloride migration test (RCMT) according to NT BUILD 492 [28], (b) water permeability in accordance with BIS 516 (2018) [29], and (c) sorptivity (initial and secondary) as per ASTM C-1585-13 [30]. The results of the durability tests are presented in Table 6.

#### 2.2.7. Strength Efficiency (SE) and Environmental Efficiency (EE)

The SE and EE of the optimized slag blend, G45, were calculated using the formulae established by Chakurkar and Guptha [15] and compared with Com-PSC.  Table 7 presents the SE, while Table 8 shows the EE.

## 3. Results and Discussions

The results of the aforementioned tests performed on all slag combinations derived from GGBFS supplementation with PSD-engineered C-OPC are thoroughly discussed in terms of their physio-mechanical performance, in comparison with Com-PSC available in the country.

### 3.1. X-Ray Fluorescence

Table 3 meticulously delineates the chemical composition of all slag blends, revealing a discernible trend: as the slag percentage increases, there is a corresponding reduction in calcium oxide content, accompanied by a proportional increase in alumina-silicates percentages. This trend is primarily attributed to the higher oxide content of silica and aluminum in GGBFS compared to C-OPC and the stimulator. In comparison with Com-PSC, calcium oxide levels were lower beyond a 45% slag supplement, whereas silica and aluminum oxide levels improved for all slag combinations.

### 3.2. PSD

**Figure 2:**
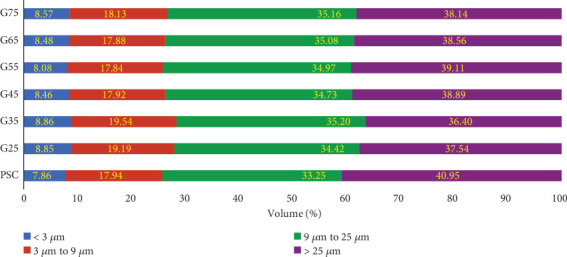
Classification of particle sizes in slag combinations and commercial PSC.

**Figure 3:**
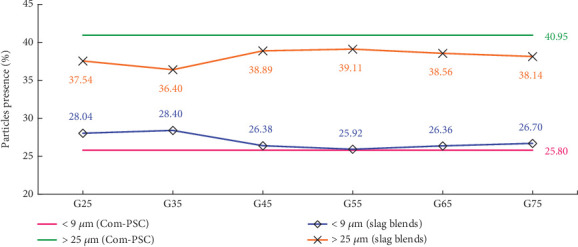
Effect of particle size variation due to PSD-engineered C-OPC in slag combinations.

In cement manufacturing, PSD is recognized as a parameter of equal importance to the chemical composition of the cement matrix. Chakurkar and Guptha [15] reported significant improvements in the compressive strength of C-OPC using innovative PSD methodologies. This study adopts the approach proposed by Chakurkar and Guptha [15] and integrates it with GGBFS. The PSD patterns for the evaluated combinations are shown in Figure 2, with distinct particle ranges presented in Figure 3, which align with the findings of Chakurkar and Guptha [15].

As shown in Figure 2, the incorporation of a stimulator in the slag combinations reduced the presence of coarser particles (> 25 *μ*m) by 4%–11%, with a corresponding increase in finer particles (<3 *μ*m) by 3%– 13% compared to the Com-PSC. The reorientation of PSD in slag combinations, resulting from the integration of PSD-engineered C-OPC with GGBFS, as opposed to Com-PSC, is illustrated in Figure 3. An increase of up to 3% in the volume of particles below 9 *μ*m is observed across all slag combinations, while a reduction of up to 5% is noted in particles above 25 *μ*m.

### 3.3. SC

**Figure 4:**
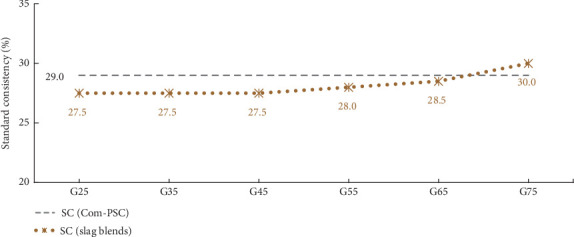
Standard consistency of slag combinations and commercial PSC.

Figure 4 presents the results of the SC test for the binary blend of S45 and GGBFS at various percentage levels. Recent studies have shown that water consistency tends to increase with higher GGBFS percentages [14]. Notably, the findings reported by Chakurkar and Guptha [15] closely align with the observations in the current study, highlighting the influence of stimulator content and its associated PSD.

Figure 4 demonstrates that up to a 45% slag replacement, there is no substantial change in the SC results; instead, the values remain relatively constant. This stability is a key factor contributing to the enhanced compressive strength of the blend. Beyond this threshold, up to a 65% slag replacement, the increase in consistency is modest and remains below the values observed for Com-PSC. However, beyond the 65% substitution level, the water requirement significantly increases and shows values above the Com-PSC.

This pronounced effect is primarily attributed to variations in the PSD. As illustrated in Figures 2 and 3, there is a considerable reduction in particles greater than 25 *μ*m, along with a corresponding increase in particles smaller than 9 *μ*m, up to the 45% slag replacement level. This distribution indicates a constant water requirement for the exothermic reaction. On the other hand, due to a marginal increase in particles below 9 *μ*m and a decrease in those above 25 *μ*m, the water demand for the hydration process increases beyond the G45 level.

### 3.4. Initial and Final Setting Times

**Figure 5:**
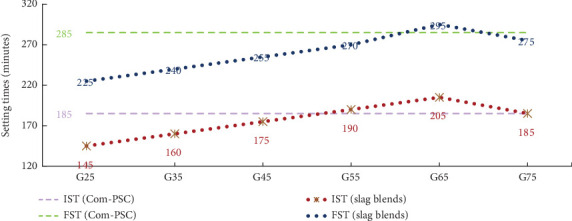
Setting times of slag combinations and commercial PSC.

Figure 5 illustrates the setting times of stimulator-assisted slag blends, which exhibit a notable trend. A continual delay in setting times is observed with an increase in GGBFS content. However, beyond a 65% blend, a significant reduction in setting times is noted. The study indicates that the optimal blending range lies between 45% and 65%, with a recommendation that blends exceeding 65% require careful consideration of setting times.

In summary, the study concludes that the addition of GGBFS in a binary binder prolongs the setting times. This delay is attributed to the slower hydration process, consequently retarding the setting times. All sets of slag combinations up to 55% replacement showed improved values compared to the commercial PSC. Importantly, none of the binary blend combinations violated the codal provisions as per BIS 455 [17], ensuring compliance with established standards.

### 3.5. Compressive Strength

Compressive strength is a critical mechanical property for both cement and cement concrete, serving as a key parameter for manufacturers. Overcoming the challenge of achieving higher strengths at an early stage has prompted the exploration of blended cements, particularly in light of sustainability concerns and the scrutiny of carbon emissions within the cement industry. Moreover, optimizing cement content while ensuring the quality of blended materials remains a significant challenge.

**Figure 6:**
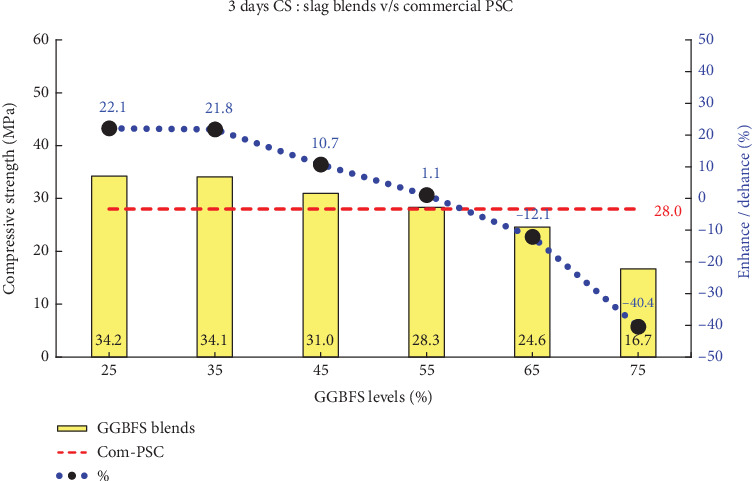
Comparison of 3-day compressive strength: slag combinations vs. Com-PSC.

**Figure 7:**
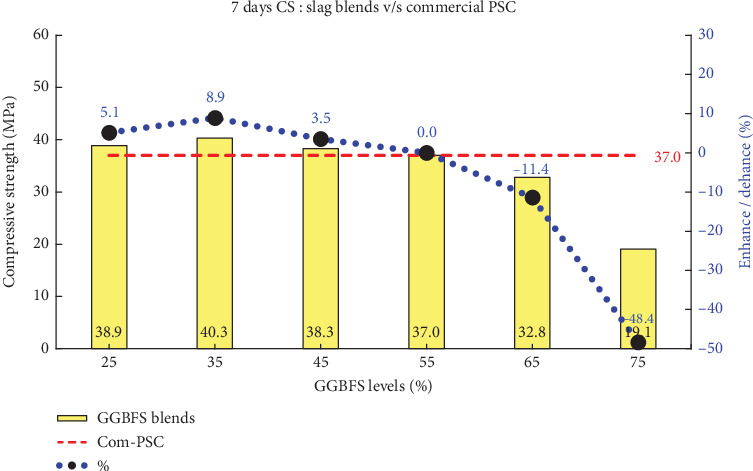
Comparison of 7-day compressive strength: slag combinations vs. Com-PSC.

**Figure 8:**
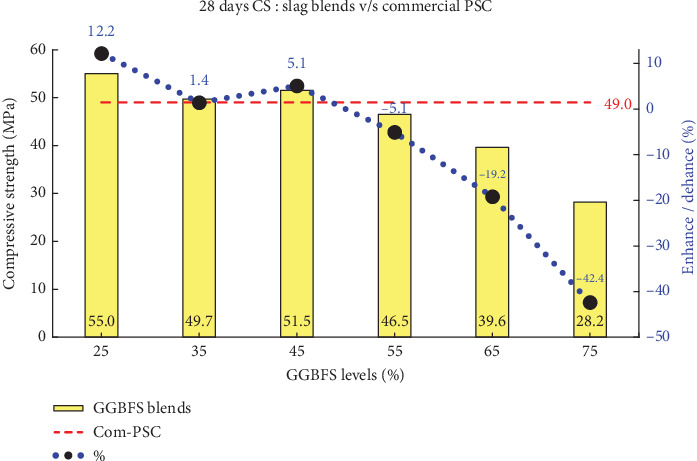
Comparison of 28-day compressive strength: slag combinations vs. Com-PSC.

This study demonstrates that PSD plays a crucial role in enhancing compressive strength at both early and later ages across various blend percentages. The comprehensive assessment of compressive strengths at intervals of 3, 7, and 28 days is systematically presented in Table 5. Figures 6, 7, and 8 illustrates the comparison of all slag combinations with Com-PSC at the respective curing ages.

It can be observed in Figure 6 that an enhancement in strength ranging from 5% to 22% is achieved in slag-blended cement with up to 50% slag replacement compared to Com-PSC at a curing age of 3 days. Similarly, as illustrated in Figures 7 and 8, strength improvements of up to 9% and 12% are observed at curing ages of 7 and 28 days, respectively, for slag replacement levels up to 50%, compared to commercial PSC, which contains 40% slag replacement. This improvement is primarily attributed to the engineered PSD of C-OPC obtained from the amalgamation of the stimulator.

Primarily, the strength enhancement at early curing ages is directly correlated with the accelerated chemical reactions within the improved fine binder particles, which, in turn, influence the overall performance of slag-blended cement. The compressive strengths for the various slag combinations were analyzed, and the observed results are presented in Table 5 for the specified curing periods: 3, 7, and 28 days.

### 3.6. SE and EE

Table 9 provides a comparative analysis of C-OPC, S45, Com-PSC, and G45, showing that G45 achieves a substantial reduction in carbon emissions to 0.447 tonnes, compared to 0.80 tonnes for C-OPC and 0.48 tonnes for Com-PSC. The correlation between strength and environmental efficiency is substantiated in Tables 7 and 8.

Table 7 shows a 1.25% improvement in the SE of the optimized slab blend, G45, compared to Com-PSC, while Table 8 highlights a 13.10% increase in environmental efficiency for G45 over Com-PSC.

## 4. Sustainability and Cost Implications

**Figure 9:**
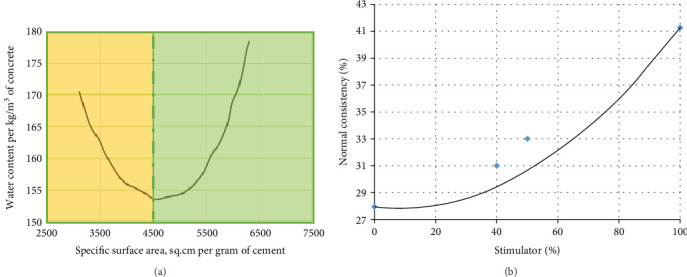
Relationship between the fineness of cement and water demand: (a) findings of Higginson on water requirements and fineness of cement in terms of specific surface area. (b) Consistency results obtained in this study.

Chakurkar and Guptha [15] indicated a slight increase (10–17%) in water requirement for consistency. However, the overall efficiency gained from reduced cement usage outweighs this increase, ensuring the economical production of high-quality cement and concrete. The study on stimulator-assisted cement demonstrates its performance as experimentally conceptualized by Higginson [31] (Figure 9a, with corresponding results in Figure 9b).

These findings highlight the potential of stimulator-assisted cement as a groundbreaking innovation in the realm of cement production, offering significant economic, environmental, and performance advantages for large-scale construction projects. The results of the current study emerge from the integration of slag blending with stimulator-assisted cement. According to Tables 10 and 11, there is a notable cost reduction of 9.20%, coupled with enhancements in overall sustainability metrics such as energy consumption and carbon emissions. A whooping saving of 19% is also achieved in concrete production, as analyzed in Table 12.

It is well documented that blended cement surpasses C-OPC in terms of durability criteria. The results illustrated in Table 6 demonstrate that stimulator-assisted blended slag cement consistently outperforms all other types of cement. These superior outcomes are largely attributed to the engineered PSD.

## 5. Conclusions

The investigation reveals distinct effects on the properties of stimulator-assisted slag combinations with engineered PSD, encompassing distribution of particle sizes, SC, setting times, and compressive strengths.

Key conclusions drawn from the study are as follows:

a. The optimized blend demonstrates minimal changes in consistency with the addition of blend percentages up to 65%. This contrasts with results reported in the literature, which emphasize the pivotal role of PSD in reducing consistency. The subsequent improvement in compressive strength over time is attributed to the lower consistency values.
b. SC shows a typical increase with the increasing percentage of GGBFS content. The findings suggest that both water and SC requirements can be effectively managed with controlled PSD.
c. IST and FST results are influenced by the normal consistency values, which reflect the sequential participation of fines. The marginal enhancement in setting times corresponding to normal consistency values provides flexibility for on-site requirements without compromising the quality of the cement.
d. The suggested blend range helps minimize the risk of cold joints in larger concrete pours.
e. GGBFS has an overall retarding effect on SC, initial setting time, and final setting time. Increases in GGBFS content intensify this retardation, which is attributed to the lower content of tricalcium aluminate (C_3_A).
f. Results underscore the dominance of C-OPC up to a 25% blend of GGBFS. Beyond 25%, GGBFS gains an advantage. All stimulator-assisted slag blends up to 65% replacement satisfy codal strength requirements at 28 days. Literature and Technical Data Sheet (TDS) support the observation that, in the absence of the stimulator, GGBFS blends fail to achieve the desired strength beyond 40%.
g. The tests performed on the slag-blended combinations accentuate the importance of stimulator incorporation, improving the contribution of particle sizes to overall cement performance. Boosted strength gain at early curing ages is attained with a low water–cement ratio. Also, improvements in setting times, including both initial and final, are observed in comparison to the Com-PSC.
h. The present work achieves an important milestone by increasing the SCM content by 25% beyond the existing commercial practices in the production of slag cement in the industry. This leads to a substantial leap towards sustainable, progressive, and eco-friendly goals.

The research findings highlight the significant potential of PSD in enhancing both initial and final compressive strength when compared to commercial slag cement, with performance observed over 365 days. These materials provide an efficient pathway for producing greener cement without increasing production costs, thereby reducing carbon footprints and contributing to a safer environment. This methodology provides a viable opportunity for cement industries to enhance the performance of slag cement. In addition, we acknowledge that the quality of slag is influenced by its source, and therefore, future research focused on developing comprehensive guidelines for optimizing slag utilization would further enrich this area of study.

## Figures and Tables

**Table 1:** Properties of C-OPC, S, and GGBFS.

	**C-OPC**	**S**	**GGBFS**
*Physical properties*			
Specific gravity	3.15	3.15	2.90
Blaine fineness, m^2^/kg	365	539	440
*Chemical properties*			
Calcium oxide, CaO (%)	57.5	59.1	37.6
Silicon dioxide, SiO_2_ (%)	20.5	20.7	34.8
Aluminum oxide, Al_2_O_3_ (%)	6.1	5.7	17.9
Ferric oxide, Fe_2_O_3_ (%)	3.3	3.8	0.7
Magnesium oxide, MgO (%)	4.2	5.6	7.8
Sulfur oxide, SO_3_ (%)	2.7	2.3	0.2
Potassium oxide, K_2_O (%)	1.4	0.9	—
Sodium oxide, Na_2_O (%)	0.1	0.2	—

**Table 2:** Fractions of S45 and GGBFS in slag combinations and Com-PSC.

**Combinations**	**Fraction (%)**		
	**C-OPC**	**S45**⁣^∗^	**GGBFS**
G25	—	75	25
G35	—	65	35
G45	—	55	45
G55	—	45	55
G65	—	35	65
G75	—	25	75
Com-PSC	60	—	40

⁣^∗^Source: Chakurkar and Guptha [15].

**Table 3:** Chemical composition of slag combinations and Com-PSC.

	**Slag combinations**						**Com-PSC**
	**G25**	**G35**	**G45**	**G55**	**G65**	**G75**	
*Chemical properties*							
Calcium oxide, CaO (%)	48.78	50.43	47.46	45.31	43.19	41.07	47.00
Silicon dioxide, SiO_2_ (%)	28.92	24.97	29.83	31.26	32.72	34.32	27.00
Aluminum oxide, Al_2_O_3_ (%)	12.48	9.53	12.26	13.55	14.78	16.43	11.00
Ferric oxide, Fe_2_O_3_ (%)	0.00	2.08	0.00	0.00	0.00	0.00	2.35
Magnesium oxide, MgO (%)	5.64	4.82	5.51	5.83	6.10	6.50	5.70
Sulfur oxide, SO_3_ (%)	3.17	2.80	2.34	2.02	1.74	1.42	2.60
Potassium oxide, K_2_O (%)	1.15	1.17	1.01	0.91	0.81	0.71	—
Sodium oxide, Na_2_O (%)	0.31	0.26	0.26	0.27	0.27	0.25	—

**Table 4:** Particle size distribution (PSD) of slag combinations and Com-PSC.

	**Particle diameter (*μ*m)**		
	**d**_10_	**d**_50_	**d**_90_
G25	3.33	18.06	54.97
G35	3.32	17.62	53.23
G45	3.47	18.96	54.94
G55	3.61	19.11	54.93
G65	3.47	18.88	53.94
G75	3.43	18.68	53.68
Com-PSC	3.67	19.78	56.65

**Table 5:** Test outcomes on slag combinations and Com-PSC.

**Powder**	**Tests**					
	**Consistency**	**Setting times**		**Compressive strength at**		
	**SC**	**IST**	**FST**	**3 days**	**7 days**	**28 days**
	**(%)**	**(min)**	**(min)**	**(MPa)**	**(MPa)**	**(MPa)**
G25	27.5	145	225	34.2 ± 1.21	38.9 ± 0.81	55.0 ± 1.59
G35	27.5	160	240	34.1 ± 1.21	40.3 ± 0.68	49.7 ± 0.25
G45	27.5	175	255	31.0 ± 0.96	38.3 ± 0.81	51.5 ± 0.49
G55	28.0	190	270	28.3 ± 0.80	37.0 ± 0.52	46.5 ± 0.35
G65	28.5	205	295	24.6 ± 1.15	32.8 ± 0.45	39.6 ± 0.97
G75	30.0	185	275	16.7 ± 0.70	19.1 ± 0.75	28.2 ± 1.53
Com-PSC	29.0	185	285	28.0	37.0	49.0

**Table 6:** Durability tests on concrete cubes.

**Tests**	**G45**	
	**M25**	**M40**
RCMT	5.84 × 10^−12^ m^2^/s	3.65 × 10^−12^ m^2^/s
Water permeability	10 mm	9 mm
Sorptivity		
Initial	0.012273 mm/s^1/2^	0.006184 mm/s^1/2^
Secondary	0.000193 mm/s^1/2^	0.000738 mm/s^1/2^

**Table 7:** Strength efficiency.

	**Com-PSC**	**G45**
28-days strength (MPa)	49.00	51.50
Cement consumption (g)	200.0	200.0
Strength efficiency (%)	24.50	25.75

**Table 8:** Environmental efficiency.

	**Com-PSC**	**G45**
28-days strength (MPa)	49.00	51.50
Carbon released (per tonne)	0.480	0.447
Environmental efficiency (%)	102.10	115.20

**Table 9:** Quantities of CO_2_ emissions from various materials.

**Materials**	**Amount of CO**_**2**_ ** released (tonnes)**
⁣^∗^1-tonne of C-OPC	0.8000
⁣^∗^CO_2_ released during “stimulator” production using 30 kWh electricity for grinding 1 tonne of C-OPC	0.0285
⁣^∗^1-tonne of Stimulator	0.8285
⁣^∗^1-tonne of S45 (i.e., 55% C-OPC+45% S)	0.8128
1-tonne of G45 (i.e., 55% S45+45% GGBFS)	55% × 0.8128 = 0.4470
1-tonne of Com-PSC	60% × 0.8000 = 0.4800

⁣^∗^Source: Chakurkar and Guptha [15].

**Table 10:** Cost analysis of cement at the production level.

		**Unit**	**Cost**
Cost of C-OPC	A	Per tonne	₹ 8000.00
Cost of C-OPC		Per kg	₹ 8.00
Cost of C-OPC		Per bag	₹ 400.00
Extra cost towards the production of the stimulator	B	Per tonne	₹ 2800.00
Cost of stimulator	A + B	Per tonne	₹ 10,800.00
Cost of stimulator		Per kg	₹ 10.80
Cost of stimulator		Per bag	₹ 540.00
Cost of S45 (i.e., 55% C-OPC + 45% stimulator)		Per kg	₹ 9.26
Cost of S45	C	Per bag	₹ 463.00
Cost of GGBFS		Per kg	₹ 4.00
Cost of GGBFS	D	Per bag	₹ 200.00
Cost of G45 (i.e., 55% S45 + 45% GGBFS)		Per kg	₹ 6.90
Cost of G45	E	Per bag	₹ 345.00
Cost of Com-PSC (from open market)		Per bag	₹ 360.00

**Table 11:** Cost reduction in cement due to enhanced efficiency.

**Parameter**	**Com-PSC**	**G45**
Compressive strength @ 28 days (MPa)	49.0	51.5
% reduction in the quantity of cement for strength at par (i.e., % increase in compressive strength)	—	5.1
Cost of cement per kg (from Table10)	₹ 7.20	₹ 6.90
Cost of cement for reduced amount of cement, per kg	₹ 7.20	₹ 6.54
Cost of cement per bag	₹ 360.00	₹ 327.00
% reduction in cost	—	9.20

**Table 12:** Cost reduction in concrete due to enhanced efficiency.

**Concrete grade**	**Cement consumed per cu.m of concrete**		**Quantity saving in cement (%)**	**Cost of concrete/cu.m**		**Reduction in concrete cost (%)**
	**Com-PSC**	**G45**		**Com-PSC (@ Rs. 360/bag)**	**G45 (@ Rs. 327/bag)**	
M25	350 (7.0 bags)	310 (6.2 bags)	11.4	2520	2027	19.6
M40	450 (9.0 bags)	400 (8.0 bags)	11.1	3240	2616	19.3

## Data Availability

The data supporting the findings of this study are available from the corresponding author upon reasonable request.
